# Maintaining mRNA Integrity during Decalcification of Mineralized Tissues

**DOI:** 10.1371/journal.pone.0058154

**Published:** 2013-03-07

**Authors:** Daniele Belluoccio, Lynn Rowley, Christopher B. Little, John F. Bateman

**Affiliations:** 1 Murdoch Childrens Research Institute, Parkville, Victoria, Australia; 2 Department of Biochemistry and Molecular Biology, University of Melbourne, Parkville, Victoria, Australia; 3 Raymond Purves Bone and Joint Research Laboratories, Kolling Institute of Medical Research, St. Leonards, New South Wales, Australia; University of Western Ontario, Canada

## Abstract

Biomineralization of the extracellular matrix occurs inappropriately in numerous pathological conditions such as cancer and vascular disease, but during normal mammalian development calcification is restricted to the formation of the skeleton and dentition. The comprehensive study of gene expression in mineralized skeletal tissues has been compromized by the traditional decalcification/fixation methods that result in significant mRNA degradation. In this study we developed a novel RNA*later*/EDTA decalcification method that protects the integrity of the mRNA in mature mouse tibial epiphyses. Furthermore, this method preserves the tissue structure to allow histological sectioning and microdissection to determine region-specific gene expression, in addition to immuno- and *in situ* histology. This method will be widely applicable to the molecular analysis of calcified tissues in various pathological conditions, and will be of particular importance in dissection of the gene expression in mouse bone and joint tissues during development and in important clinical conditions such as arthritis.

## Introduction

Degenerative joint disease is a major clinical problem and the molecular study of gene expression networks in joint tissues such as articular cartilage and subchondral bone is of fundamental importance to our understanding of disease mechanisms and to identify potential biomarkers and possible therapeutic targets [1]. The central pathological feature of osteoarthritis is the progressive destruction of articular cartilage and this tissue has been the focus of numerous studies, not least because noncalcified cartilage can be recovered readily by surface dissection of mature human joint tissues recovered during surgery for joint replacements, or post-mortem. However, the use of predominantly noncalcified cartilage because it is readily able to be dissected from the joint surface without decalcification, ignores that fact that osteoarthritis is a disease of the whole joint, including in particular the pathological changes in the underlying calcified cartilage and subchondral bone.

To address the inherent shortcomings in human-based osteoarthritis cartilage studies which rely largely on analysis of end-stage disease, several rodent models have been developed [2] to allow harvesting of joint tissues during the onset and progression of the disease for mRNA expression analysis. The fact that the models develop with a reproducible time course and the age, sex, exercise and genetic background of the animals can be controlled makes them ideal to study the pathological events in osteoarthritis progression. However, the study of degeneration of the calcified tissues of the skeletally mature rodent joint requires first decalcification using either rapid acid-based methods or prolonged exposure to chelating agents, both of which limit downstream analysis to histology and immunohistology after fixation. Since current decalcification methods have been shown to result in significant RNA degradation [3], [4], direct mRNA expression analysis has not be possible on calcified cartilage or other calcified joint tissues.

To overcome this limitation we developed a novel decalcification approach that preserves the integrity of the mRNA in mature mouse tibial epiphyses and allows microdissection of cartilage regional zones for mRNA expression profiling. We show that this procedure also conserves the histological morphology which allows *in situ* hybridization analysis of mRNA and immunohistochemical analysis of protein localization. While this method has been developed for the analysis of cartilage expression in joint degeneration, it will have wide applicability in the transcriptomic analysis of other calcified tissues of the skeleton during normal development and in the pathological calcification that occurs in numerous human diseases such as cancer and vascular disease.

## Materials and Methods

### Isolation of Tibial Epiphyses

The mouse studies were approved by the Murdoch Childrens Research Institute Animal Ethics Committee (Approval #A672). Male C57BL6 mice were obtained from an inbred SPF colony, where animals were housed in 12 hour light dark cycles and received irradiated pelleted food and autoclaved water *ad libitum*. Mice were sacrificed 10 weeks of age and the tibial epiphyses, which included the articular surface, calcified deep cartilage and subchondral bone were isolated from both hind limbs of all mice, dissected free of the other joint tissues, washed briefly in PBS and either snap frozen or subjected to decalcification within 15 minutes of death.

### Decalcification

The tibial epiphyses were randomly assigned to one of three decalcification methods. One group of epiphyses were decalcified using conventional ethylene-diamine-tetraacetic acid (EDTA) chelation, where each tibiae was immersed in 5 ml of 20% EDTA/Tris-HCl, pH 7.4, and gently agitated for 72 hrs at 4°C. Following decalcification tibiae were snap frozen and imbedded in OCT and stored at −80°C. Other epiphyses were decalcified using 5 ml of 10% EDTA in RNA*later* (Ambion) at either pH 5.2, or pH 9.2, for 72 hrs at 4°C with gentle agitation. To produce the RNA*later*/EDTA solutions, solid EDTA was added to RNA*later* which was adjusted to pH 9.2 with NaOH to dissolve the EDTA. To produce RNA*later*/EDTA at pH 5.2, the pH was adjusted by addition of HCl. Following decalcification tibiae were briefly washed with RNAse-free PBS (2×5 ml, 1 min), snap frozen and embedded in OCT and stored at −80°C.

### Histology, *In Situ* Hybridization and Immunohistochemisty

Serial 10 µm coronal cryo-sections were taken from the OCT-embedded tibiae. Isolated sections from each tibia were stained with toluidine blue/fast green using routine procedures [5]. *In situ* hybridization was performed using either digoxigenin (DIG)-UTP labeled [6] or [^35^S]CTP labeled [7] antisense and sense (control) RNA probes. To prepare the probes, RT-PCR was performed using specific primer sets for *Col2a1* and *Prg4* (Table 1) and the PCR products were cloned into pGEM T-Easy vector (Promega). The vector was linearized and transcribed in the presence of DIG-UTP (DIG RNA Labeling Kit; Roche) or [^35^S]CTP (Amersham) using the appropriate RNA polymerase (T7 or SP6; Roche). DIG-labeled probes were hybridized to tissue sections overnight at 65°C, washed under stringent conditions as previously described [6], blocked and incubated overnight at room temperature with DIG anti-Fab fragments (1∶1000; Roche Diagnostics). After washing in blocking solution, sections were stained using nitro blue tetrazolium chloride (NBT) and bromo-4-chromo-3-indolyl phosphate (BCIP) in alkaline phosphatase staining buffer until a strong specific signal was detected [6]. Images were captured by bright field microscopy. [^35^S]CTP labeled probes were hybridized overnight at 58°C and then washed under stringent conditions [7]. Autoradiography was performed using Hypercoat LM-1 emulsion (Amersham) and exposed for 3 weeks prior to development. The slides were counterstained with Mayer’s hematoxylin (Sigma) and the hybridized radio-labeled probes were visualized using a dark field equipped Leitz diaplan microscope (Leitz) using a red filter and a bright field picture was captured by using a blue filter.

**Table 1:** PCR primers.

*In situ* hybridization probes			
Gene	Forward primer (5′-3′)	Reverse Primer (5′-3′)	Size
***Col2a1***	AACGTCCAGATGACTTTCCTC	ATTTTGCAGTCTGCCCAGTTC	994 bp
***Prg4***	TGAAGATGCAGATGGAGGTG	GTCTGGAAAGGTCCAACAGC	654 bp
**Quantitative PCR**			
**Gene**	**Forward primer (5′-3′)**	**Reverse Primer (5′-3′)**	**Size**
***Co2a1***	ACACTTTCCAACCGCAGTCA	GGGAGGACGGTTGGGTATCA	76 bp
***Rpl10***	TTGAAGACATGGTTGCTGAGA	AGGACCACGATTGGGGATA	74 bp

For immunohistochemistry, frozen sections were thaw-mounted onto Superfrost plus glass slides (Menzel). The sections were fixed in HistoChoice® Tissue Fixative (Amresco) for 15 minutes at 4°C then washed in 3 changes of PBS. Sections were treated with 30µg/ml Proteinase K in 50 mM Tris-HCl, pH 6.0, 5 mM CaCl_2_ at 37°C for 30 min, and 0.2% hyaluronidase (bovine, type IV; Sigma) for 1 h at 37°C. After blocking with 1% goat serum in 1% BSA (w/v) in PBS overnight at 4°C, the sections were incubated with mouse anti-collagen II antibody (1∶1000; Clone 2B1.5, Thermo Scientific) or affinity purified rabbit anti-human collagen VI polyclonal antibody (1∶2000; Fitzgerald Industries) and visualized by immunofluorescence with Alexa-Fluor 488 conjugated secondary antibody (1∶200; Molecular Probes). Sections were counterstained with DAPI to visualize nuclei.

### Microdissection, RNA Extraction and Quality Assessment

Serial 7-µm coronal cryo-sections of EDTA or RNA*later*/EDTA decalcified samples mounted on RNase-free SuperFrost slides (Menzel) were fixed in 70% ethanol, washed in RNase-free water, and dehydrated in 70%, 95%, and 100% ethanol for one minute each and air-dried. Slides were then immobilized on an inverted microscope (Leica) and articular cartilage was dissected by using an ophthalmic scalpel (Feather) that was attached to a xy-stage by a custom-made device that allowed rotational movement in the z-axis [7]. In other experiments Laser Capture Microdissection (Arcturus) was used to isolate microdissected cartilage zones (data not shown). The tissue was then harvested into RNase-free micro-centrifuge tubes and total RNA was extracted using TRIzol (Invitrogen) and isolated using an RNeasy kit (Qiagen) including an on-column DNase I digestion. RNA was also similarly extracted from EDTA or RNA*later*/EDTA decalcified whole tibiae, pulverized using a liquid nitrogen-cooled tissue grinder. The purity and concentration of the RNA was measured using a Nanodrop ND-1000 spectrophotometer (Thermo Fisher Scientific) and integrity of the RNA samples was determined by capillary electrophoresis with a Bioanalyzer 2100 (Agilent), using a Series II RNA 6000 Pico Kit (Agilent), according to the manufacturer’s specifications.

### Quantitative PCR

RNA isolated from EDTA or RNA*later*/EDTA decalcified whole tibiae (500 ng total RNA) was reverse-transcribed as per the manufacturer’s protocol in a 20µL reaction using the Transcriptor High Fidelity cDNA Synthesis Kit (Roche, Germany). This reaction was incubated at 50°C for 30 minutes, 85°C for 5 minutes. Quantitative PCR (qPCR) reactions containing 2.5 ng cDNA were prepared to 5µL in 384 well PCR plates (Roche) using the LightCycler® 480 SYBR Green I Master (Roche) as per the manufacturer’s instructions and run on a LC480 II thermo cycler (Roche). *Col2a1* and *Rpl10* primer details are provided in Table 1. Cycling conditions for qPCR reactions were; Pre-incubation, 1 cycle of 95°C (5 minutes, 4.8°C/s ramp rate); Amplification, 45 cycles of 95°C (10 seconds 4.8°C/s ramp rate), 60°C (30 seconds 2.5°C/s ramp rate), 72°C (10 seconds, 4.8°C/s ramp rate); Melting Curve, 1 cycle of 95°C (5 seconds 4.8°C/s ramp rate, 65°C (1 minute 2.5°C/s ramp rate), 97°C (0.11°C/s ramp rate). Analysis of the run file from the cycler was done using the Roche LightCycler 480 Software release 1.5.0 to calculate C_p_ values for each sample.

## Results and Discussion

**Figure 1:**
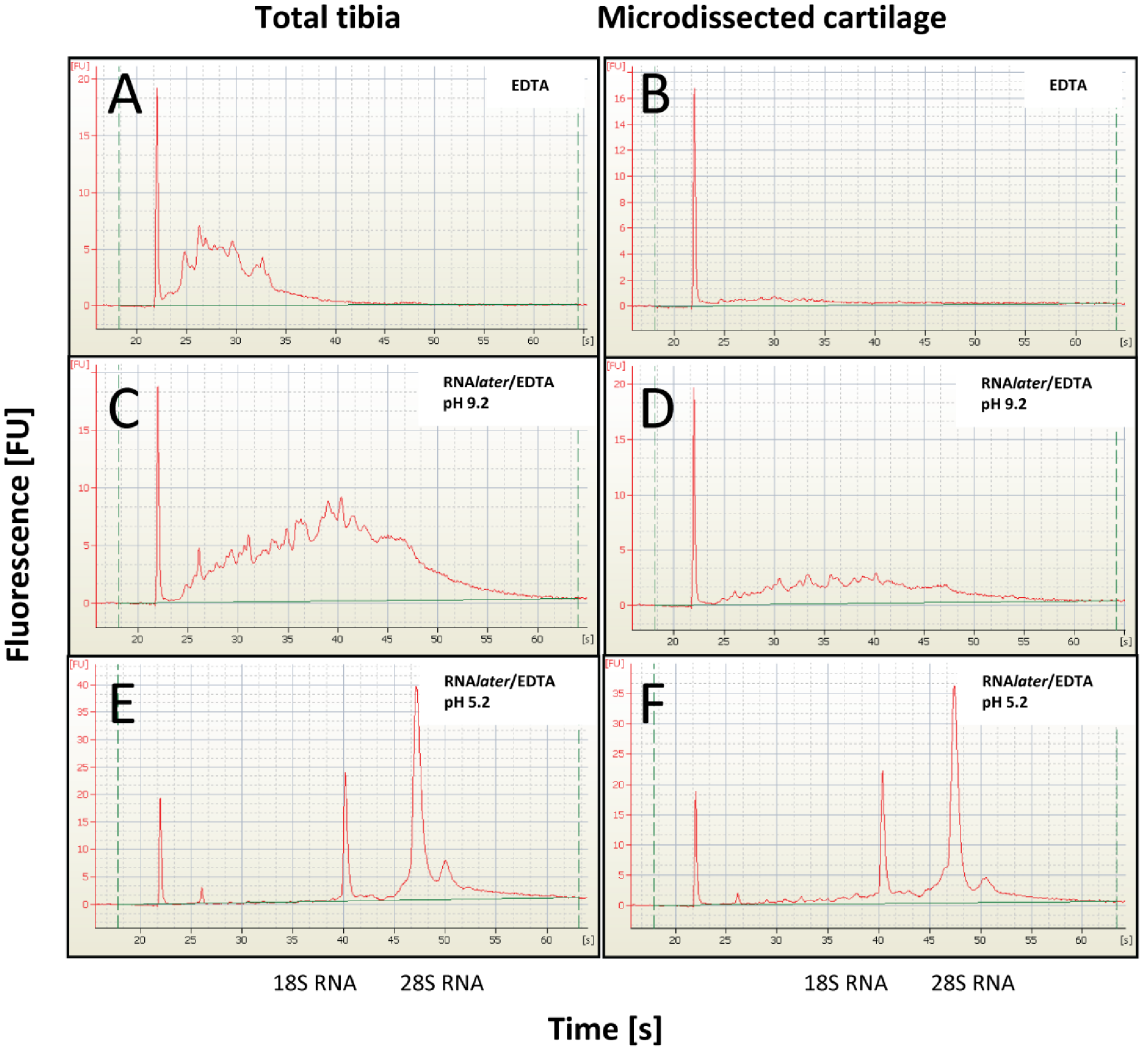
Analysis of RNA integrity. Microcapillary electrophoresis of total RNA isolated from whole tibial epiphysis (A, C, E) or cryosections of tibiae (B, D, F) decalcified with 0.5 M EDTA (A, B), RNA*later*/EDTA at pH 9.2 (C, D) or RNA*later*/EDTA at pH5.2 (E, F).

We found that standard EDTA decalcification procedures severely impacted on the quality of the RNA extracted from both whole tibiae (Fig. 1A) or from microdissected tibial cartilage (Fig. 1B), as determined by microcapillary electrophoresis. In both cases there was little or no 18S or 28S RNA reflected in the low RNA Integrity Numbers (RIN) of 2.3 and 1.0, respectively. These data were consistent with recent studies demonstrating the deleterious effects of current decalcification methods on RNA integrity [3], [4].

**Figure 2:**
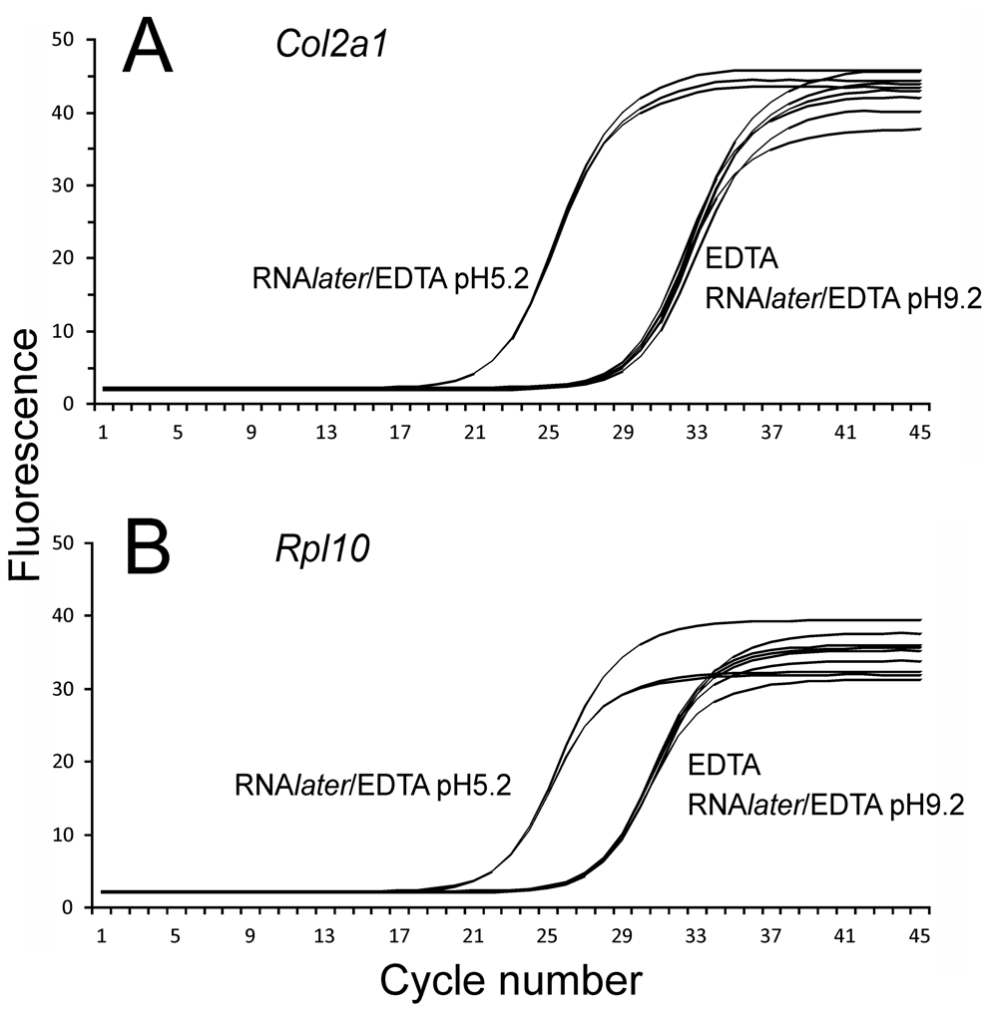
Quantitative PCR. Total RNA extracted from tibiae decalicified with 0.5 M EDTA, RNA*later*/EDTA at pH 9.2 or RNA*later*/EDTA at pH5.2 was analyzed for *Col2a1* (A) or *Rpl10* (B) mRNA transcripts by q PCR using Sybr Green. The machine output (Roche LightCycler 480 II) of fluorescence at each PCR cycle number is shown.

**Figure 3:**
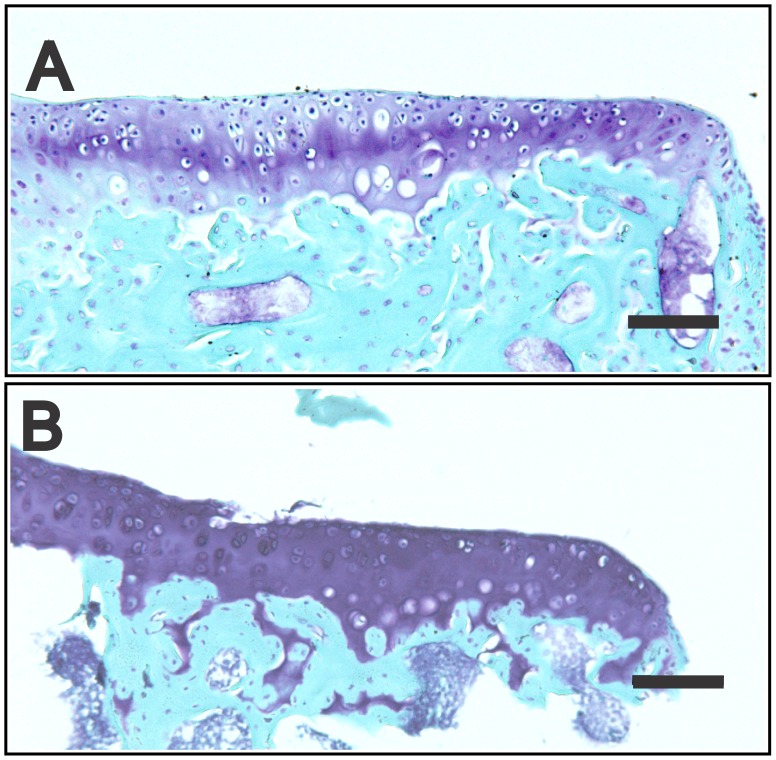
Cartilage morphology after EDTA or RNA*later*/EDTA decalcification. Tibial epiphyses were decalcified for 72 hrs at 4°C with 0.5 M EDTA (A) or RNA*later*/10% EDTA at pH 5.2 and cryosections were stained with toluidine blue/fast green. The medial tibial plateau is shown. Cartilage morphology and aggrecan staining is preserved in the RNA*later*/10% EDTA, pH 5.2 decalcified samples. Scale bar = 100 µm.

In an attempt to overcome this serious problem with the need to decalcify mineralized tissues prior to any downstream mRNA profiling studies, we combined the RNA stabilizing agent RNA*later* (Ambion) with EDTA in the decalcification protocol. Using decalcification times of 72 hrs (at 4°C), equivalent to standard 20% EDTA procedures, we first decalcified the tibiae in RNA*later* containing 10% EDTA at pH9.2 (Fig. 1C, D) which was the adjusted pH required to allow initial EDTA dissolution. These results showed very little protection of the RNA from degradation, with RINs of 2.7 (whole tibia) and 2.3 for the microdissected samples. In an attempt to further improve RNA quality we reduced the pH of the RNA*later*/EDTA to pH5.2 (Fig. 1E, F) which corresponded to the usual working pH of RNA*later*. With this decalcification method RNA was completely protected from degradation with RIN values of 10 and 9.2. While this method provides an important new approach to obtaining high quality mRNA for expression analysis, it was important to determine if the RNA*later*/EDTA decalcification was compatible with other downstream analyses such as qPCR, histology, *in situ* mRNA expression analysis and immunohistochemical detection of protein localization. For qPCR, equal amounts of RNA isolated from EDTA or RNA*later*/EDTA decalcified whole tibiae was reverse-transcribed and analyzed by qPCR for the expression of *Col2a1* and the house-keeping gene *Rpl10* (Fig. 2). For both genes EDTA and RNA*later*/EDTA, pH 9.2 decalcification performed similarly with threshold cycles of amplification (C_p_) of ∼29.3 cycles for *Col2a1* (Fig. 2A) and ∼27.4 cycles for *Rpl10* (Fig. 2B). In contrast, when RNA was extracted after RNA*later*/EDTA, pH 5.2 decalcification, the detection of both gene transcripts was far more robust with C_p_ of ∼22.0 cycles for *Col2a1* (Fig. 2A) and ∼22.1 cycles for *Rpl10* (Fig. 2B), representing an increased abundance of amplifiable target transcript of approximately ∼150-fold for *Col2a1* and approximately ∼40-fold for *Rpl10*. This qPCR data confirms the protection of RNA degradation by RNA*later*/EDTA, pH 5.2 shown in Figure 1E. Histological staining of the medial tibial plateau demonstrated that the RNA*later*/EDTA decalcification maintained the tissue morphology as demonstrated by Toluidine blue staining (Fig. 3B). Furthermore, RNA*later*/EDTA decalcification preserved the toluidine blue-staining proteoglycans in the tissue sections much better than conventional EDTA decalcification (Fig. 3A). Aggrecan loss during EDTA decalcification has been previously reported [5], and thus decalcification of unfixed/uncrosslinked tissues in RNA*later*/EDTA provides superior preservation of the cartilage aggrecan, and possibly other associated cartilage proteins.

**Figure 4:**
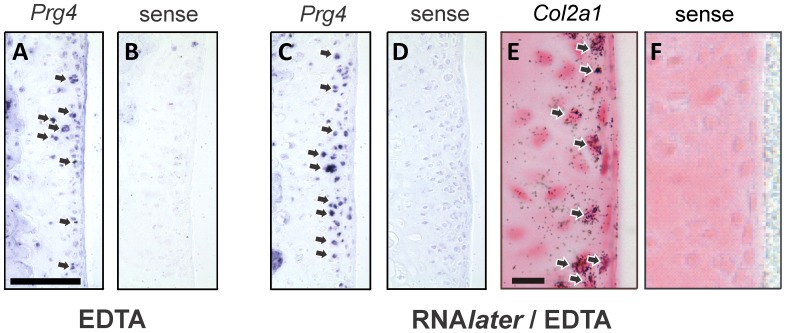
*In situ* hybridization for *Prg4* and *Col2a1*. Cryosections from tibia decalcified with EDTA (A,B) or RNA*later*/EDTA at pH5.2 (C–F) were hybridized with DIG-labeled *Prg4* antisense (A,C) or sense (B,D) RNA probes, and against ^35^S-labeled *Col2a1* antisense (E) or sense (F) RNA probes for *Col2a1*. Arrows show representative regions of target gene mRNA expression. Scale bars = 100 µm (A–D), 10 µm (E, F).

The preservation of the tissue morphology coupled with mRNA protection afforded by the method was evident in the ability to conduct *in situ* mRNA expression analysis of the cartilage (Fig. 4). We examined the expression of *Prg4* which encodes lubricin, a glycoprotein involved in joint lubrication that is expressed in cartilage by only the superficial articular chondrocytes [8], and *Col2a1* which is expressed throughout the articular cartilage. Both *Prg4* (Fig. 4C) and the more highly expressed *Col2a1* (Fig. 4E) are readily detected in the RNA*later*/EDTA samples. Although not quantifiable, the *Prg4* signal in the RNA*later*/EDTA samples was stronger than that seen in the samples decalcified by EDTA alone (Fig. 4A), consistent with the protection of RNA from degradation in the RNA*later*/EDTA samples (Fig. 1).

**Figure 5:**
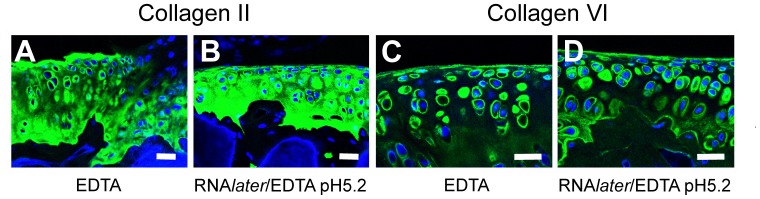
Fluorescent immunohistochemistry of articular cartilage. Collagen II (A, B) and collagen VI (C, D) protein localization was unaffected by RNA*later*/EDTA, pH 5.2 decalcification (B, D) compared to conventional EDTA decalcification (A, C). Using both methods collagen II can be seen in both the pericellular and extracellular matrix, while collagen VI is predominantly localized to the pericellular matrix. DAPI was used as a nuclear stain. Scale bars = 50 µm.

In addition to mRNA integrity in the decalcified tissue sections, immunohistochemical staining for cartilage proteins was possible. We demonstrated the cartilage localization of collagen II (Fig. 5B) and collagen VI (Fig. 5D) was preserved compared to the conventionally EDTA-decalcified samples (Fig. 5A, C) and it is important to note that the pericellular collagen VI extracellular matrix characteristic of chondrocytes was also intact after RNA*later*/EDTA treatment (Fig. 5D).

In this study we have developed a simple method for decalcification of mineralized tissues that protects RNA integrity for downstream expression analysis by quantitative PCR, microarray expression profiling or *in situ* hybridization, while preserving tissue morphology for histology and immunohistochemistry. In addition to these advantages, this method does not affect the protein composition of the tissue (data not shown), allowing parallel proteomic studies to be conducted, providing the opportunity for a systems biology level analysis of the biology of calcified tissues. In these studies we have applied this technique to mouse tibial epiphyseal cartilage, which has immediate application to the study of joint degeneration and arthritis mechanisms. However, there are other important developmental contexts, and pathological situations such as vascular calcification and cancer, where this approach should be of value.
